# Regulation of N-type calcium channels by nociceptin receptors and its possible role in neurological disorders

**DOI:** 10.1186/s13041-022-00982-z

**Published:** 2022-11-24

**Authors:** Emanuelle Sistherenn Caminski, Flavia Tasmin Techera Antunes, Ivana Assis Souza, Eliane Dallegrave, Gerald W. Zamponi

**Affiliations:** 1 Graduate Program in Health Sciences, Laboratory of Research in Toxicology (LAPETOX), Federal University of Health Sciences of Porto Alegre, Porto Alegre, RS Brazil; 2 Department of Clinical Neurosciences, University of Calgary, Calgary, AB Canada; 3 Hotchkiss Brain Institute, University of Calgary, Calgary, AB Canada

**Keywords:** Anxiety, Learning, ORL-1, Opioid receptor, Pain, G protein-coupled receptor

## Abstract

Activation of nociceptin opioid peptide receptors (NOP, a.k.a. opioid-like receptor-1, ORL-1) by the ligand nociceptin/orphanin FQ, leads to G protein-dependent regulation of Cav2.2 (N-type) voltage-gated calcium channels (VGCCs). This typically causes a reduction in calcium currents, triggering changes in presynaptic calcium levels and thus neurotransmission. Because of the widespread expression patterns of NOP and VGCCs across multiple brain regions, the dorsal horn of the spinal cord, and the dorsal root ganglia, this results in the alteration of numerous neurophysiological features. Here we review the regulation of N-type calcium channels by the NOP-nociceptin system in the context of neurological conditions such as anxiety, addiction, and pain.

## Structure and physiological roles of N-type calcium channels

Voltage-gated calcium channels (VGCCs) mediate calcium influx upon the membrane depolarization, regulating many physiological processes, such as neurotransmitter release, hormone secretion, muscle contraction, and gene expression [1]. The dysfunction of these channels has been linked to a wide range of pathological conditions including pain, epilepsy, migraine, night blindness, cardiac arrhythmias, and congenital deafness among many others [2, 3], making them important pharmacological targets [4]. VGCCs have been classified based on their electrophysiological and pharmacological profiles. They can be separated into low-voltage activated (LVA) calcium channels, which include the T-type calcium channels (Cav3.1, Cav3.2, and Cav3.3), or high-voltage activated ones (HVA), including N-type (Cav2.2), R-type (Cav2.3), P/Q-type (Cav2.1), and L-type (Cav1.1, Cav1.2, Cav1.3, and Cav1.4) channels [5, 6]. With the exception of Cav1.1 which is skeletal muscle-specific, these channels are all expressed in the brain, but show varying albeit overlapping distributions and support specific neurophysiological roles [1]. Cav2 channels operate predominantly at presynaptic terminals where they are responsible for fast synaptic transmission [7], whereas Cav1.3 and Cav1.4 channels can both be expressed at ribbon synapses (where they support features such as auditory and visual synaptic transmission [8, 9]; with Cav1.2 and Cav1.3 being expressed as well on cell bodies and dendrites of many types of central nervous system neurons [10]. All calcium channels contain a pore forming Cavα1 subunit that is obligatory to obtain a functional channel and which are comprised of four transmembrane domains that are connected by cytoplasmic linker regions and flanked by cytoplasmic N- and C-terminal regions [1, 5]. HVA channel Cavα_1_ subunits co-assemble with ancillary Cavα_2_δ and Cavβ subunits in a 1:1:1 stoichiometry [11]. These subunits help promote plasma membrane expression and regulate the biophysical and pharmacological features of the channel [12, 13]. The mammalian genome expresses four different types of Cavβ and four types of Cavα_2_δ subunits, with the latter being post-translationally cleaved, relinked via a disulfide bond, and then attached to the outer leaflet of the plasma membrane via a GPI anchor [14, 15]. In contrast, Cavβ subunits are cytoplasmic and attach to the Cavα_1_ subunit at the linker between transmembrane domains I and II. With additional diversity arising from alternative splicing events, the existence of multiple ancillary subunits gives rise to a wide range of different calcium channel complexes [16, 17]. The cryo-EM structure of Cav2.2 (and other) calcium channels has been solved [18–21] and has provided insights into the structural basis of channels function and pharmacology.

Cav2.2 calcium channels are expressed predominantly at presynaptic nerve terminals where they (along with Cav2.1 channels) are critically involved in the release of neurotransmitters [22, 23]. Their expression is primarily restricted to neurons, with wide distribution in the brain as well as in peripheral sensory afferents. Cav2.2 channels are potently inhibited by peptide toxins isolated from different types of marine fish hunting cone snails such as ω-conotoxin MVIIA (*Conus magus*) and ω-conotoxin GVIA (*Conus geographus*) whose selectivity of action on these channels has helped elucidate key roles of these channels in neurophysiological processes [24, 25]. Intrathecal delivery of such toxins mediates potent analgesic effects [26, 27], consistent with a key role of these channels in neurotransmission between sensory afferents and neurons in the spinal dorsal horn. Case in point, a synthetic version of ω-conotoxin MVIIA (a.k.a. Ziconotide or Prialt) is FDA approved for the treatment of refractory cancer pain [28]. Additional insights into the physiological roles of Cav2.2 channels have been gained from genetic models such as knockout mice lacking these channels, from small organic inhibitors of Cav2.2 channel activity, and from molecules that modulate post-translational modification of these channels. Mice deficient in Cav2.2 show hyposensitivity to different pain modalities [29–31], consistent with the data obtained with pharmacological inhibition. These mice also show increased vigilance [32], hyper-aggression [33], reduced anxiety levels [29], and appear to exhibit a reduction in the symptoms of alcohol withdrawal [34]. The latter is also consistent with the action of small organic Cav2.2 inhibitors [35–37]. A link between Cav2.2 channels and reward-seeking was highlighted by studies that target the interaction of Cav2.2 with collapsin mediator protein 2 (CRMP2) which is involved in regulating Cav2.2 channel expression at the plasma membrane [38, 39]—interfering with this regulatory pathway alters reinstatement of cocaine seeking [40]. Pharmacological inhibition of Cav2.2 has been shown to have anxiolytic effects [41, 42], and this may be related to an inhibition of GABAergic synaptic transmission in the basolateral amygdala and prefrontal cortex [43]. On the other hand, it was shown that alternative splicing of exon 37 of Cav2.2 enhances responses to aversive stimuli via alteration of glutamatergic signaling [17]. Deletion of Cav2.2 channels either directly, or by depletion of its ancillary Cavβ3 subunit has been linked to impairment of memory [44, 45]. Finally, delivery of the Cav2.2 inhibitor ω-conotoxin GVIA directly into the brain has been shown to cause depression-like behavior in rodents [46], although it should be noted that depression is more commonly associated with altered L-type calcium channel activity. Overall, these findings indicate that Cav2.2 channels mediate important physiological roles and that modulation of the activity of these channels is expected to have consequences that affect behaviors such as anxiety and pain responses.

### Modulation of Cav2.2 channels by NOP receptors

Cav2.2 channels are subject to modulation by a wide range of G protein coupled receptors (GPCRs) [47]. This includes the nociceptin opioid receptor (NOP, previously called the opioid receptor-like 1 receptor (ORL-1), which was first described by Mollereau et al. [48] and added as a fourth member of the opioid receptor family of µ-, κ-, and δ-opioid receptors [49, 50]. However, the NOP receptor is not activated by any of the known endogenous opioids that act on classical opioid receptors, but instead by the endogenous agonist nociceptin/orphanin FQ [51, 52]. The crystal structure of the NOP receptor reveals a typical 7 transmembrane helix topology with an additional short helix contained within the proximal C-terminus [53].

**Fig. 1:**
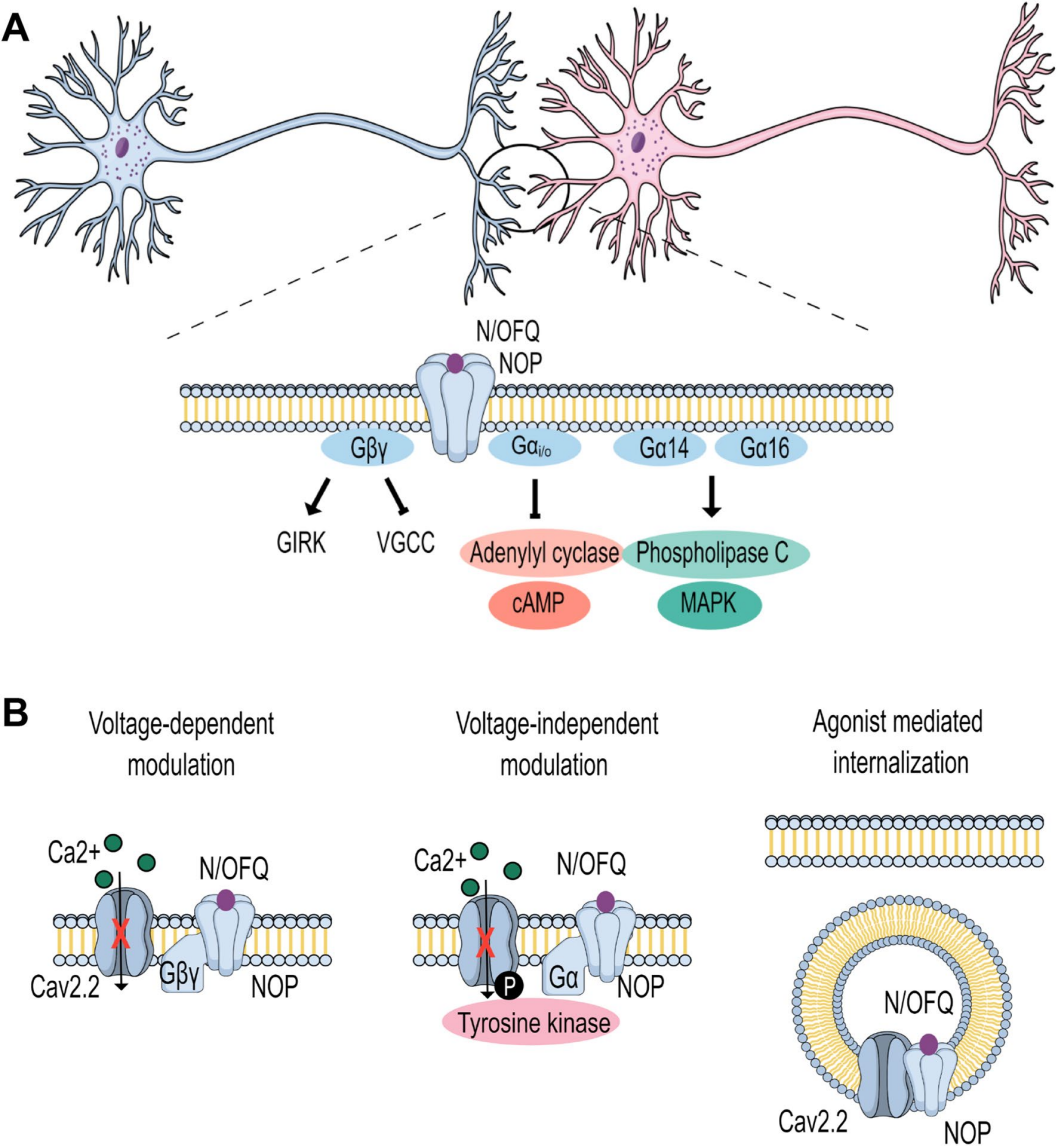
Key aspects of NOP receptor signalling. **A**. NOP receptors at synaptic terminals. Activation of NOP by N/OFQ triggers several G protein dependent signalling pathways. **B**. Activation of NOP receptors triggers Gβγ mediated voltage-dependent inhibition of Cav2.2 channels, as well as Gα/tyrosine kinase dependent voltage independent modulation. Due to the formation of signalling complexes, prolonged agonist application triggers the co-internalization of NOP receptor Cav2.2 channel complexes

Like other G protein-coupled receptors (GPCRs), the activation of NOP by nociceptin triggers the exchange of GDP for GTP on the Gα subunit, causing its dissociation from the Gβγ dimer. The dissociated subunits then proceed to act on different signaling pathways and effector systems. The NOP receptor couples primarily to Gα_i/o_, inhibiting adenylyl cyclase and thus cyclic adenosine monophosphate (cAMP) production [54]. In addition, activation of NOP receptors activates phospholipase C and MAP kinase activity via coupling to Gα_14_ and Gα_16_ [55]. The dissociated Gβγ subunits, in addition to regulating various intracellular signaling cascades, activate G protein-coupled inwardly rectifying potassium (GIRK) channels [56], and inhibit VGCCs [47], thus reducing neuronal excitability and neurotransmitter release (Fig. 1A).

Specifically, Gβγ subunits inhibit members of the Cav2 calcium channel family (Fig. 1B) by directly interacting with the Cavα_1_ subunit at its cytoplasmic N-terminal and domain I-II linker regions [57–59], with Cav2.2 channels being inhibited more potently than Cav2.1 [60–62]. Direct Gβγ modulation of Cav2.2 is sensitive to the membrane potential and can be reversed by the application of a depolarizing pre-pulse or by trains of action potentials—it is therefore referred to as voltage-dependent inhibition [63–65]. The extent of G protein modulation is also dependent on the Cavβ subunit [66–68] and critically dependent on the Gβ isoform [61, 69], thus allowing receptors to mediate a wide range of inhibitory voltage-dependent processes. Many G protein-coupled receptors also cause voltage-independent modulation of channel activity, most likely via the activation of protein kinases and receptor tyrosine kinases (Fig. 1B). This type of inhibition is insensitive to membrane depolarizations and has also been observed with NOP receptors [70].

Inhibition of Cav2.2 calcium channels by NOP receptors has been described in both neurons and heterologous expression systems [70–77]. In addition to the classical G protein inhibition, the C-terminus of the NOP receptor physically interacts with the Cav2.2 C-terminus, allowing it to control channel density at the plasma membrane through enhanced forward trafficking by possibly promoting ER export, and via channel/receptor co-internalization after prolonged agonist exposure [70, 78, 79] (Fig. 1B). This physical interaction also allows for a tonic inhibition of Cav2.2 channels by Gβγ in the absence of an agonist, presumably due to the receptor’s constitutive activity. Another potential avenue by which the NOP can modulate Cav2.2 channels is via heterodimerization with other opioid receptor family members. NOPs form heterodimer complexes with all of the other three opioid receptors, and the prolonged activation of µ-opioid receptors triggers the internalization of Cav2.2 channels, but only in the presence of NOP receptors [80].

A number of different ligands of NOP receptors have been identified and they are not considered to be opioids. As already noted above, the archetypal agonist of the NOP receptor is nociception—a 17-amino acid peptide that is structurally related to dynorphin A [54]. A wide range of synthetic NOP receptor agonists have been described (for review, see [81]) and have been examined in preclinical and clinical studies of anxiety and pain. Conversely, a wide range of NOP receptor antagonists have been developed and tested in animals and human subjects in phase I and II studies for the treatment of cognitive impairment, depression, and addiction [81, 82]. This includes LY2940094 (aka BTRX-246040) which appears to be effective in the treatment of depression and well tolerated [83]. Mixed agonists of NOP and opioid receptors such as AT-121 and cepranopol have also been explored as potential systemically active pain therapeutics including in phase III clinical testing [84, 85]. The NOP receptor is widely distributed in the central and peripheral nervous systems [86]. In the brain, it is expressed in regions that are involved in the processing of pain, learning, memory, food intake, and fear [86–88], consistent with the above pharmacological studies. Given the widespread expression of VGCCs, they are co-expressed with these receptors in many areas of the brain. Below, we will highlight the role of the NOP-N/OFQ system in neuro-physiological processes that are known to rely on Cav2.2 channels.

### NOP receptors as modulators of pain

NOP receptors are expressed in the dorsal and ventral horns of the spinal cord which integrate sensory processing [89]. NOP receptor expression is enhanced in the spinal cord in rodent models of persistent inflammatory and neuropathic pain [90–92] and there is an upregulation of plasma concentrations of nociceptin in chronic pain patients [93]. Intrathecal delivery of nociceptin mediates analgesia in rodent models of pain [94–97], as do non-peptide agonists [98–100]. In non-human primates, systemic or intrathecal injection of NOP agonists causes antinociception [101–105]. These analgesic effects are likely mediated by an inhibitory action on presynaptic Cav2.2 channels expressed in sensory afferents (note that NOP receptors are expressed on both A and C fibers [79, 86, 106], along with possible activation of postsynaptic GIRK channels). In addition, NOP receptor activity has been shown to modulate the excitability of spinal interneurons and superficial layer neurons projecting to the brain [107–109].

Mice lacking the NOP receptors show normal nociceptive thresholds, only slightly reduced locomotor activity, and normal immune responses, but compromised hearing ability [110]. These mice however also show alterations in morphine tolerance. Like NOP receptors, activation of μ-opioid receptors by morphine or DAMGO also inhibits Cav2.2 channel activity [111]. While NOP receptors are intrinsically resistant to the μ-opioid specific agonist morphine, they appear to affect morphine tolerance via crosstalk with μ-opioid receptors. It was shown that mice lacking NOP receptors exhibit resistance to morphine tolerance without alterations in the acute effects of morphine [112, 113], whereas mice with morphine tolerance exhibit upregulation of NOP receptors [113]. Along these lines, NOP receptor activation diminishes the development of morphine tolerance in rats and blocks morphine-induced place preference [114, 115], although this concept has been challenged [116]. Other studies have reported an antagonistic effect of NOP receptor activation on opioid-induced analgesia [117, 118]. In addition, increased NOP expression is seen after prolonged morphine exposure [113, 119, 120]. Finally, bi-functional ligands of NOP and μ-opioid receptors help prevent adverse effects associated with μ-opioid receptor activation [121–123]. To what extent this crosstalk is mediated by selective actions via Cav2.2 calcium channels remains to be determined, but the notion that μ-opioid receptors, NOP receptors, and Cav2.2 channels can form signaling complexes [80] hints at the possibility that converging signaling by these two receptors on Cav2.2 as a common target may play a role.

While NOP receptors mediate antinociceptive actions at the spinal level likely by inhibition of spinal excitatory neurons and synaptic transmission at peripheral afferent terminals [124], they appear to exhibit pronociceptive effects at the supraspinal level, as observed after injection of nociceptin into the brain [51, 52]. It is not entirely clear how these pronociceptive effects occur at the cellular and molecular level. One possibility may be an inhibitory effect on stress-induced analgesia that is known to be modulated by NOP receptors [125–127]. Indeed, there is evidence that NOP receptor activation contributes to the occurrence of allodynia and hyperalgesia in post-traumatic stress disorders (PTSD) [128], with NOP antagonists reversing this type of pain behavior [129]. It has been suggested that at supraspinal sites, the activation of NOP receptors may inhibit neuronal activity via activation of GIRK channels in brain loci that are relevant for nociceptive transmission such as the periaqueductal gray region and dorsal raphe nucleus [124], but exactly how this modulation causes pro-nociceptive effects remains to be worked out.

### Role of NOP receptors in anxiety

Anxiety is marked by excessive fear (and avoidance) in response to perceived threatening situations and even in the absence of true danger [130]. NOP receptors are highly expressed in neurons and synaptic terminals of brain areas that are implicated in anxiety and depression [131]. This includes the amygdala, the nucleus accumbens (NAc), the hippocampus, the hypothalamus, the periaqueductal gray, the ventral tegmental area (VTA), and some of the prefrontal regions among others [132–134]. The medial prefrontal cortex (mPFC) exerts inhibitory control over amygdala activity, preventing an improper expression of emotion, but in anxiety disorders, this control becomes imperfect and consequently triggers aberrant amygdala activation through dysfunction of GABAergic and glutamatergic transmission [135–138]. NOP activation produces presynaptic inhibition of VGCCs, and thus the release of glutamate in the lateral amygdala [139, 140]. NOP agonists delivered orally or via intraperitoneal injections have revealed anxiolytic effects in rodents [141–144]. Little is known about the biochemical pathways by which NOP agonists induce anxiolytic effects but they may involve serotonin availability and changes in firing rates in the dorsal raphe nucleus (DRN, which projects to serotonergic axons of the amygdala), and concomitantly, by counteracting anxiogenic CRF actions into the bed nucleus of *stria terminalis* [145]. It should be noted that some studies have, however, reported anxiogenic responses to nociceptin, and the reason for these discordant findings is unclear [146, 147].

Prenatal ethanol exposure (PEE) induces an anxiety-like phenotype in adult and adolescent rats [148], and this is frequently used as an animal model to study anxiety. This results in the induction of neuronal hyper-excitability by augmenting glutamatergic transmission, which does not appear to be attenuated by GABAergic inhibition in the basolateral amygdala [149, 150]. NOP activation via intracerebroventricular administration of nociceptin reportedly inhibits associated anxiety behaviors [151]. In this model, there is an alteration of dopaminergic neuron morphology [152], along with a reduction of NOP gene expression [153], and an increase in the expression of the precursor of nociceptin (ppN/OFQ) [151]. Although the authors did not find any alterations of the NOP-nociceptin pathway in the amygdala, alterations of upstream inputs may lead to altered amygdala activity, thus perhaps contributing to the anxiety phenotype.

Altogether, anxiolytic-like effects can be observed with the delivery of nociceptin and synthetic agonists into the brain. Cav2.2 channels are also known to play a role in anxiety and are a potential pharmacological target for anxiolytics [41, 154, 155]. Indeed, as noted earlier, inhibition or depletion of Cav2.2 channels has been shown to mediate anxiolytic effects in mice [29, 156], and therefore a putative inhibition of these channels by activation of NOP would be expected to result in similar outcomes. It is however unclear precisely in which brain structures and neuronal subtypes of NOP receptor regulation of Cav2.2 channels may take place in the context of anxiety.

### NOP receptors and stress

The fear/anxiety neuro-circuitry overlaps with the neuro-circuitry that regulates stress responses [130]. The amygdala integrates information from cortical and thalamic sensory inputs, to generate fear and anxiety-related behavioral outputs [157], with fear conditioning models demonstrating an increase in synaptic strength in the lateral amygdala (LA) and the PFC [158–160]. PTSD involves an over-reactivity of the HPA axis, decreased inhibitory signaling by GABA, and increased excitatory neurotransmission. This is associated with increased activity in emotion-processing brain regions, such as the limbic system (hippocampus, amygdala, PFC). Overall, a major characteristic of PTSD is impaired fear extinction, following exposure to a traumatic event [161]. These responses against chronic stress lead to altered NOP receptor signaling which may contribute to an increase in corticosterone levels [145]. In PTSD experimental models, corticosterone levels, as well as nociceptin and NOP expression are increased in different brain regions and the cerebrospinal fluid, and consequently, NOP antagonists protect against molecular and behavioral deficits [162, 163]. Much of the underlying mechanisms appear to involve alterations in L-type calcium channel activity [156, 164–170]. However, given that Cav2.2 channels are present at synaptic junctions in the HPA axis, and are typically inhibited by NOP receptor agonists, it is possible that these channels play a detrimental role in fear extinction during PTSD, in addition to their potential pronociceptive effects that were discussed earlier. On the other hand, it is interesting to note that compounds such as topiramate which block R-type and N-type channels appear to show some promise in treating PTSD [171]. Clearly, more work is needed to elucidate the potential role of Cav2.2 channels in PTSD and its modulation by the NOP receptors.

### NOP receptors as targets for addiction

Dopamine participates in drug-seeking reward behavior by modulating the activity of the mesolimbic pathway [172]. The dopaminergic axons in the ventral tegmental area (VTA) project to downstream regions such as the nucleus accumbens, the nucleus of the stria terminalis, and the lateral hypothalamus [173]. Nociceptin and NOP receptors are densely expressed in the VTA where they regulate dopaminergic transmission [174, 175]. This occurs by disinhibition of GABAergic control over dopaminergic neurons, such that NOP agonists mediate suppression of dopamine release [115, 176, 177]. Genetic deletion or pharmacological inhibition of NOP receptors in rats was shown to inhibit nicotine-motivated behaviors [178]. Along these lines, alcohol reward-seeking behavior was shown to be inhibited upon NOP receptor antagonism or deletion [179–184]. This fits with observations that nociceptin and NOP receptor expression are aberrantly increased in many brain regions of Marchigian Sardinian alcohol-preferring rats, including the central amygdala [185]. However, as with the role of NOP receptors in anxiety, there are also several studies that show that NOP agonists may in fact inhibit reward-seeking behavior related to alcohol and cocaine [186–188]. The reason for this discrepancy is not entirely clear, and a recent review article suggested the possibility of some NOP agonists acting as functional antagonists [163]. This then makes it difficult to predict how NOP receptors modulate the reward circuitry by putative actions on calcium channels.

L-type channels have been extensively implicated in reward-seeking behavior and are known to be dysregulated during states of addiction [155, 189–192]. But there is also evidence that N-type calcium channels play a role, as Cav2.2 knockout mice exhibit reduced ethanol consumption [34]. Along these lines, the mixed N-type/T-type calcium channel inhibitor NP078585 reduced alcohol-induced intoxication and reinstatement, but failed to do so in mice lacking Cav2.2. This indicates that its primary target was indeed the N-type channel [35] and altogether suggests that the Cav2.2 channel is a potential target for the treatment of alcoholism [36]. While an inhibitory action of NOP agonists on Cav2.2 channels would be consistent with some of the results discussed above, the fact that NOP antagonists can also attenuate reward-seeking behavior suggests that Cav2.2 channels are not the exclusive effector of NOP receptors in the context of addiction.

### Role of NOP receptors in other neuropsychiatric conditions

Khan et al. [193] suggested a potential role of the NOP system in schizophrenia. Some indications that support this concept are derived from experiments involving a resident-intruder test—an animal model of aggression that is thought to reflect certain symptoms of bipolar disorder, schizophrenia, or PTSD. In this model, systemic injection of NOP antagonists had little effect, but the aggressive behavior of mice that were treated with NOP agonists was greatly increased [194]. The observed effects were similar to those seen with those of para-chlorophenylalanine, an inhibitor of serotonin synthesis [194]. An equivalent increase in aggressive behavior was seen in Cav2.2 null mice, or upon microinjection of a Cav2.2 channel inhibitor into the dorsal raphe nucleus of wild-type mice [33]. Hence, it is tempting to speculate that the aggressive behavior observed with NOP receptor agonists may have involved the inhibition of Cav2.2 channels. This might fit with the idea that Cav2.2 null mice also presented with an increase in serotonin levels in the hypothalamus. It is important to note that a recent study has shown that P/Q-type calcium channels may also play a role in aggressive behavior [195]—a Cav2 channel subtype that is also inhibited by NOP receptors.

Modeling bipolar-like behavior through the administration of psychostimulants affects several neurotransmitter systems, and thus it is difficult to attribute precise cellular and molecular mechanisms that underlie the resultant behavioral phenotype [196]. In mouse models of methylphenidate-induced manic-like behavior, the synthetic full NOP agonist Ro 65-6570 attenuated drug-induced hyperlocomotion, but did not affect spontaneous locomotor activity, whereas NOP receptor antagonists had no effect [197]. Interestingly, methylphenidate mediated similar effects in WT and NOP receptor null mice, indicating that the establishment of the hyperlocomotion does not per se involve a dysregulation of the NOP receptor system [197]. It has been shown that N-type and P/Q-type channel inhibitors prevent methylphenidate-induced locomotion [198, 199], potentially by inhibiting dopamine release. This is thus consistent with a mechanism by which NOP receptor activation mediates stimulant-induced hyperlocomotion by inhibition of Cav2.2 channels.

### NOP receptor function in movement disorders

Central vestibular neurons are important for processing motion-related multisensory signals and their transformation into motor commands [200]. Participation of NOP receptors and nociceptin in vestibular afferent excitability has been demonstrated via the locomotor performance of rats after traumatic head injury. Such rats exhibit motor deficits and increased levels of nociceptin in the afferent vestibular nuclei, as well as an increase in NOP receptor expression in brain regions that are associated with the vestibular function [201, 202]. The behavioral and functional deficits that occur following a traumatic brain injury also correlate with the elevated NOP expression in brain regions that are associated with vestibular function, including the motor cortex, the dorsal striatum, and the vestibular nuclei [76]. In cultured rat vestibular afferent neurons, nociceptin inhibits HVA calcium currents (principally, those carried by Cav2.2), without modifying LVA channels, and this inhibitory action occurs via voltage-dependent Gβγ modulation [76]. This fits with the idea that blockers of Cav2.2 channels are neuroprotective during traumatic brain injury [203].

In Parkinson's disease, the degeneration of the dopaminergic neurons of the substantia nigra (SN) pars compacta affects the entire basal ganglia network [204, 205], leading to hyperactivity that is sustained by enhanced glutamatergic inputs [206, 207]. In addition to the basal ganglia, dopamine depletion in the cortical and subcortical motor areas results in the upregulation of the nociceptin-NOP system in the SN, and a down-regulation in the striatum, and this may contribute to associated motor dysfunction [208, 209]. 6-hydroxydopamine (6-OHDA) hemilesioned rats exhibit an elevated expression and release of nociceptin into the SN [210] thereby causing motor deficits. There are conflicting results from pharmacological studies in Parkinson’s disease models. On one hand, exogenous nociceptin or synthetic NOP agonists, when administered systemically, attenuated dyskinesias [211, 212]. However, there was a biphasic effect on motor function, where low doses improved Parkinson-like symptoms, and higher doses disrupted motor coordination and global behavior [212]. Conversely, NOP antagonists also produced antiparkinsonian effects [213, 214] by ameliorating akinesia, bradykinesia, and overall gait problems at low doses, but they inhibited motor activity at intermediate concentrations. Interestingly, these different doses differentially regulated glutamatergic and GABAergic signaling in the SN and the ventromedial thalamus [215]. While the precise contributions of VGCCs to this NOP-dependent regulation of excitatory and inhibitory neurons remain to be determined, there is evidence that 6-OHDA mediates a PKA-dependent upregulation of Cav2.2 channels in the SN [216]. Hence, a putative inhibition of Cav2.2 channels would be consistent with a protective effect of NOP agonists.

## Conclusion

**Fig. 2:**
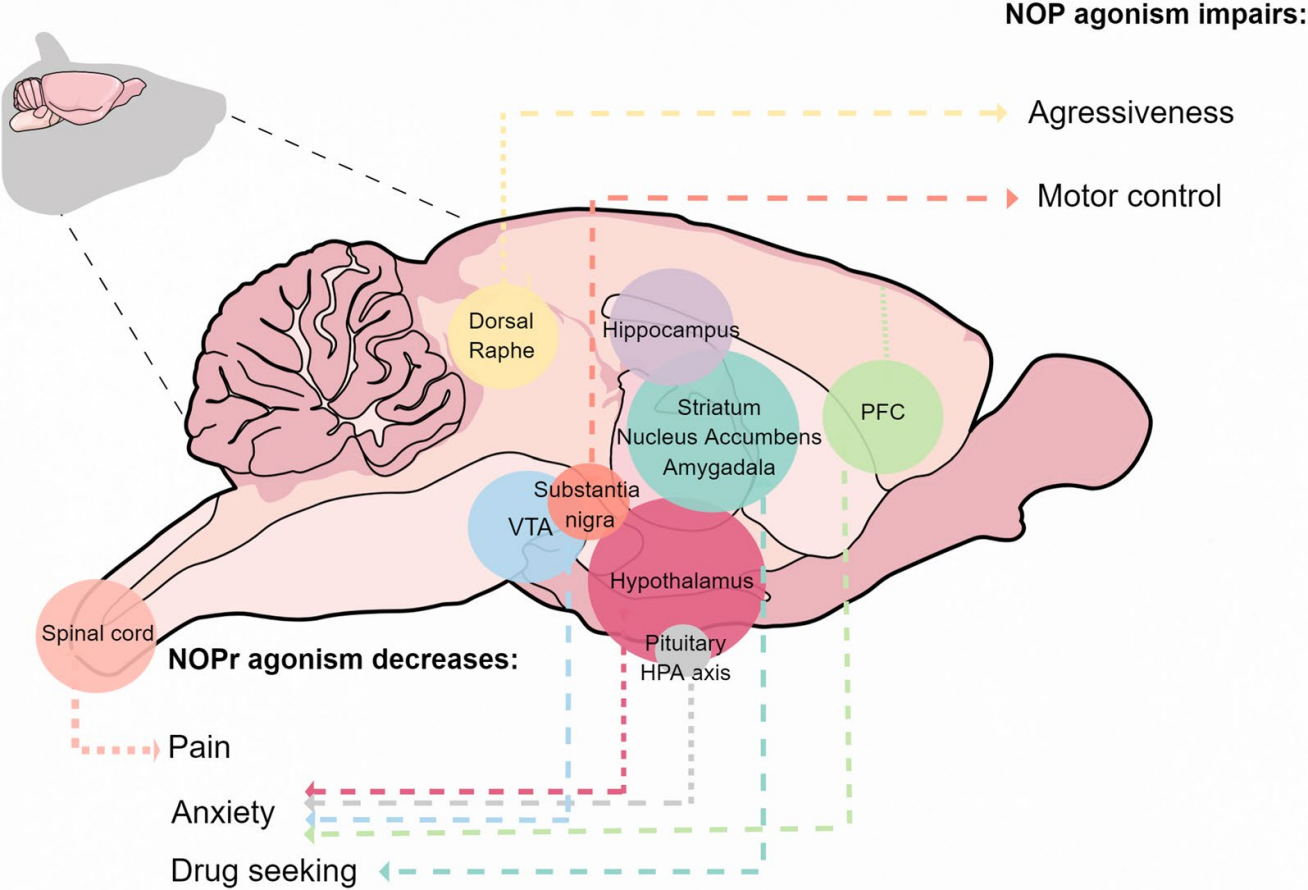
Graphical overview of key physiological roles of NOP receptors vis a vis a putative action on Cav2.2 channels

The nociceptin-NOP pathway is expressed widely in the peripheral and central nervous system and dysregulated in a number of neurological and neuropsychiatric conditions. A key target of NOP receptor activation are Cav2.2 channels which are typically inhibited upon receptor activation, but whose forward and reverse trafficking is also regulated by NOP receptor association with the channel complex. In the afferent pain pathway, there is a tight correlation between Cav2.2 channel activity and NOP receptor activation in the context of pain signaling (Fig. 2). In conditions such as anxiety, reward-seeking, and motor control the importance of Cav2.2 channels as downstream targets of NOP receptors is more tenuous (Fig. 2). While actions on Cav2.2 channels are consistent with what is known about this VGCC subtype, NOP receptors activate a plethora of downstream signaling cascades that are expected to have complex effects on neurons, glial cells, and neuronal circuits. Future studies involving the activation of these receptors in Cav2.2 null mice will ultimately provide deeper insights into putative linkages.

## Data Availability

Not applicable.

## Abbreviations

NOP: Nociceptin opioid peptide
GABA: Gamma-aminobutyric acid
PKA: Protein kinase A
HVA: High voltage-activated
LVA: Low voltage-activated
VTA: Ventral tegmental area
SN: Substantia nigra
PTSD: Post-traumatic stress syndrome
PFC: Prefrontal cortex
GIRK channel: G protein-coupled inwardly rectifying potassium channel
VGCC: Voltage-gated calcium channel
CRMP2: Collapsin mediator protein 2
SN: Substantia nigra
6-OHDA: 6-Hydroxydopamine
